# Polymorphic beams and Nature inspired circuits for optical current

**DOI:** 10.1038/srep35341

**Published:** 2016-10-13

**Authors:** José A. Rodrigo, Tatiana Alieva

**Affiliations:** 1 Universidad Complutense de Madrid, Facultad de Ciencias Físicas, Ciudad Universitaria s/n, Madrid 28040, Spain

## Abstract

Laser radiation pressure is a basis of numerous applications in science and technology such as atom cooling, particle manipulation, material processing, etc. This light force for the case of scalar beams is proportional to the intensity-weighted wavevector known as optical current. The ability to design the optical current according to the considered application brings new promising perspectives to exploit the radiation pressure. However, this is a challenging problem because it often requires confinement of the optical current within tight light curves (circuits) and adapting its local value for a particular task. Here, we present a formalism to handle this problem including its experimental demonstration. It consists of a Nature-inspired circuit shaping with independent control of the optical current provided by a new kind of beam referred to as polymorphic beam. This finding is highly relevant to diverse optical technologies and can be easily extended to electron and x-ray coherent beams.

The ability of light to exert a force on objects along its propagation direction, known as radiation pressure, is a well-understood phenomenon. This was first conjectured by Kepler in 1619 to explain why a comet’s tail (dust) points away from the sun. The first laboratory demonstrations of the radiation pressure force were reported in 1901 by Lebedev^1^ and Nichols^2^. Today, the radiation pressure is understood in the context of light-matter interaction as a consequence of the conservation of momentum during absorption and scattering of photons. As in other areas of science, the invention of the laser prompted renewed interest in the radiation pressure for optical manipulation of micro/nano-particles^3^,^4^,^5^,^6^,^7^,^8^,^9^, atom cooling^10^,^11^,^12^,^13^,^14^, material processing and cleaning^15^,^16^, etc. Interestingly, only two decades ago, it has been found that the phase of a laser beam can redirect part of the radiation pressure yielding transverse optical forces suited for manipulation of small particles. Indeed, the rotation of micro-particles induced by the familiar Gaussian optical vortex^17^,^18^,^19^,^20^,^21^,^22^ is a well-known manifestation of such transverse forces, which, however, are not restricted to the particular case of vortex beams. This kind of transverse forces is proportional to the optical current^23^ defined as **j**(**r**) = *I*(**r**)∇Φ(**r**), where *I*(**r**) and Φ(**r**) are the intensity and phase distributions of the beam with **r** = (*x*, *y*) and ∇ being the position vector and the gradient in a transverse plane, correspondingly.

To efficiently exploit the transverse forces governed by the optical current, this has to be confined into well-defined circuits with form and size easily tailored to the considered application. Such circuits for optical current correspond to high intensity gradient light curves, where the phase can be independently prescribed in a large variety of configurations providing a control of the current flow along the circuit. The combined use of high intensity and phase gradients, for example, allows for improving laser micromachining tools^24^. Moreover, three-dimensional (3D) high intensity gradients of the beam yield additional optical forces responsible for stable 3D trapping of dielectric particles while the phase gradient forces can drive their transport along the circuit^25^,^26^. Note that transverse optical forces associated to the phase gradient of a focused laser beam along a line and circle^25^ as well as along other closed and open curves^26^ have been proved suitable for 3D transport of dielectric micro-particles. These conditions cannot be fulfilled by the usually applied Gaussian beams and require the use of suitably structured diffraction-limited beams. Another important requirement is the ability to create complex circuit shapes tailored to the considered application. Fortunately, Nature has evolved many inspiring solutions to design problems. Indeed, the curved circuits can be described by an elegant expression known as Superformula, which was found by J. Gielis^27^ in the study of biological and other natural forms: shapes of plants, micro-organisms (e.g.: cells, bacteria and diatoms), small animals (e.g.: starfish), crystals, etc. The Superformula gives the radius of the curve


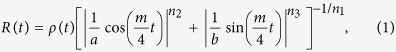


as a function of the polar angle *t*, where the real numbers in **q** = (*a*, *b*, *n*_1_, *n*_2_, *n*_3_, *m*) are the design parameters of the curve and *ρ*(*t*) is a non-periodic function of *t* required for the construction of asymmetric and spiral-like curves (e.g.: *ρ*(*t*) ∝ *e*^*αt*^ or *ρ*(*t*) ∝ *t*^*α*^). For *ρ*(*t*) = *ρ*_0_ and **q** = (1, 1, 1, 1, 1, 0), where *t* ∈ [0, 2*π*], a circle with radius *R*(*t*) = *ρ*_0_ is obtained while for other values of **q** a variety of closed polygons of different symmetry are easily generated. Note that the Superformula has also been used in the design of 3D dielectric lens antennas^28^ and to describe complex shapes of metamaterials^29^ and nanostructures^30^.

Here we introduce the concept of polymorphic beam that fulfills the aforementioned requirements. It can be focused into a diffraction-limited light curve described by the Superformula yielding optical current circuits with the following key properties: i) high intensity gradients, ii) diversity of forms with inherent biomimicry created in a practical way, iii) independent phase gradient control, iv) arbitrary design of the optical current along the circuit. This makes the focused polymorphic beam a multi-functional tool of high technological interest. In particular, the expected applications include: Single-shot laser lithography, micro-machining (e.g.: drilling and marking)^24^,^31^, photo-fabrication of structures for tissue engineering scaffolds^32^,^33^,^34^ or other sophisticated constructs^35^,^36^,^37^, transport of particles along programmed trajectories^25^,^26^ required for drug delivery, rheology, creation of colloidal motors and study of collective particle dynamics, to name a few.

## Results

### Description of a polymorphic beam

The complex field amplitude of a polymorphic beam is written as


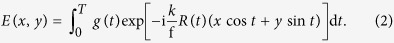


The function *g*(*t*) is a complex valued weight (with dimension of electric field) of the plane waves comprising the beam and f is a normalization constant. The parameter *T* stands for the maximum value of the azimuthal angle *t*, where *k* = 2*π*/*λ* with *λ* being the light wavelength. The radius *R*(*t*) given by Eq. (1) varies according the curve that can be either closed (*T* = 2*π* and constant *ρ*(*t*)) or open. To create the light curve (optical circuit), the polymorphic beam is Fourier transformed:


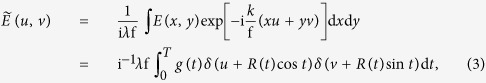


by using a convergent lens of focal length f. Taking into account the *δ* –function properties:


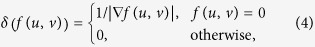


where 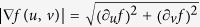, we derive that the complex field amplitude 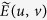 is described by the 2D curve written in parametric form as **c**(*t*) = (*u*(*t*), *v*(*t*)), with *u*(*t*) = −*R*(*t*)cos*t* and *v*(*t*) = −*R*(*t*)sin*t*. The field amplitude distribution along the curve is given by 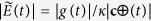, where: 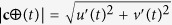 
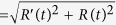
 with **c**′(*t*) = d**c**/d*t*, and *κ* = *L*/*λ*f with 
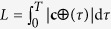
 being the curve length.

For the analysis of the optical current it is convenient to write the complex function *g*(*t*) in the form


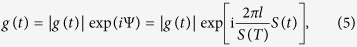


with *S*(*t*) being an arbitrary real function describing the phase variation along the curve. We underline that the parameter *l* defines the phase accumulation along the entire curve. For closed curves the phase accumulation is 2*πl* and *l* corresponds to the vortex topological charge: 
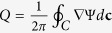
, see for example^38^. The direction of the phase gradient coincides with the curve tangent **v = **d**c/**|d**c**| and then ∇Ψ = **v** · dΨ/d**c**, where the phase derivative is given by


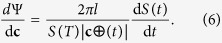


Thus, the optical current is expressed as


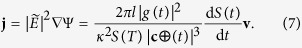


Therefore, *R*(*t*) defines the form of the circuit while *g*(*t*) prescribes the optical current along it.

In general, the value of the optical current can be modified by changing the intensity distribution or phase gradient. However, in the most of applications it is preferable to maintain uniform intensity distribution along the circuit, that corresponds to |*g*(*t*)| = *E*_0_*κ*|**c**′(*t*)|, in which case the optical current is written as


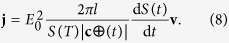


We recall that the phase function *S*(*t*) is arbitrary and can be independently specified. For example, the function


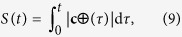


which prescribes an uniform phase distribution along the curve **c**(*t*), can be applied to create circuits with constant optical current: 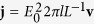.

Thus, the polymorphic beam is a perfect tool for creating arbitrary optical current, governed by *g*(*t*), confined inside an independently designed circuit with form and size given by *R*(*t*).

### Non-diffractive beams as a particular case of the polymorphic beam

For the simple case of constant radius of the curve: *R*(*t*) = *R* and *T* = 2*π*, the Eq. (2) is reduced to the well-known Whittaker’s integral^39^ describing non-diffractive beams. Indeed, for 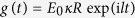, a Bessel beam^40^,^41^,^42^ that focuses into a ring-like vortex beam^25^ of radius *R* with topological charge *l* is created. The intensity and phase distributions are uniform along the ring due to |*g*(*t*)| = *E*_0_*κ*|**c**′(*t*)| = *E*_0_*κR* and *S*(*t*) = *t*, respectively. This is easy to demonstrate by considering polar coordinates *x* = *r*cos*θ* and *y* = *r*sin*θ* as it follows: The plane waves of Eq. (2) are exp[−i*κ*(*x*cos*t* + *y*sin*t*)] = exp(−i*κr*sin(*θ* − *t*)), and the resulting polymorphic beam corresponds to the helical Bessel beam of order *l*:


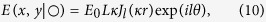


where


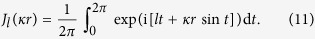


**Figure 1:**
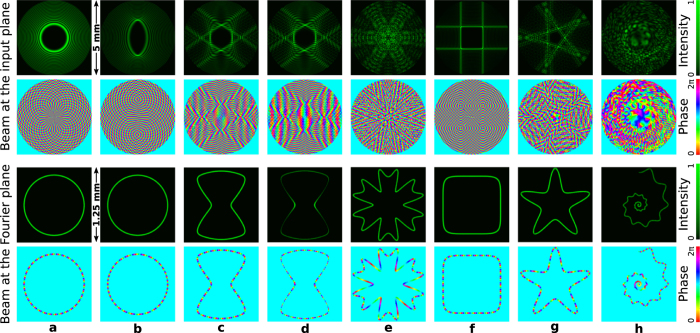
Intensity and phase distributions of the polymorphic beam (*λ* = 532 nm and f = 30 cm) at the input (top panel) and at the Fourier plane (bottom panel) for different curve shapes. (**a**,**b**) Circle of radius *R* = 0.5 mm, (**c**,**d**) sandglass, (**e**) rose, (**f**) modified-square, (**g**) starfish, and (**h**) spiral. The modulus of the function *g*(*t*) is chosen so that the intensity distribution along the optical circuits is uniform (|*g*(*t*)| ∝ |**c**′(t)|) except for (**d**) where 
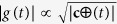
. The phase of *g*(*t*) is governed by *l* = 34 and *S*(*t*) which is uniform (Equation (9)) for (**a**), (**c**), and (**d**). While *S*(*t*) is given by the expressions 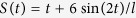 for (**b**) and Eq. (12) with *α* = 2 for (**e**–**h**). Thus, in these examples the optical current is constant for (**a**) and (**c**) whereas variable otherwise.

The top panel of Fig. 1(a) shows the intensity and phase of the helical Bessel beam 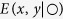 with charge *l* = 34. The corresponding focused beam (at the Fourier plane), displayed in the bottom panel of Fig. 1(a), reveals the circle of radius *R* where the wavevectors of the plane waves lie. On the other hand, the term *S*(*t*) given by Eq. (9) yields an uniform vortex phase distribution over the circuit. By maintaining constant the intensity distribution along the circle and varying the phase function 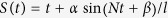 in Eq. (5), the so-called modulated vortices (which are non-diffractive in our case) are obtained^43^. The non-uniformity of the phase correspondingly changes the beam intensity in the direct domain creating *N*–fold Lissajous intensity patterns. For example, the modulated vortex described by the phase function 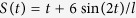 with *l* = 34 yields an elliptic-like pattern as displayed in the first row of Fig. 1(b). In spite of the fact that the circular shape of the beam and its uniform intensity distribution are preserved in the Fourier plane, the phase Ψ is non-uniform along the circle due to the term 6 sin (2*t*), see bottom panel of Fig. 1(b). We underline that while the purpose of the introduction of the modulated Gaussian vortices was the reconfiguration of the beam intensity distribution in the direct domain, the non-uniform phase distribution along the circle in the Fourier domain is indeed more important. It provides a modulated optical current expressed by 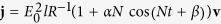 yielding variable forces exerted over particles that can be interesting for optical tweezers applications.

### Versatile shaping of the intensity and phase along circuits

The intensity and phase governing the optical current can be also designed for non-circular curves in distinct configurations. To illustrate this fact and the ability to set different optical currents for the same circuit shape, let us first consider a *sandglass* curve corresponding to **q** = (0.9, 10, 4.2, 17, 1.5, 4) in the Superformula Eq. (1). For example, the optical current in the case of Fig. 1(c) is uniform because both intensity and phase are so, whereas it is variable in Fig. 1(d) due to the non-uniform intensity created by using 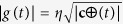. This non-uniformity of the intensity along the sandglass circuit is responsible for variation of the optical current: **j** = *κ*^−2^*η*^2^2*πl*(*L*|**c**′(*t*)|)^−1^**v**. We recall that the intensity and phase are independently prescribed along the curve and therefore it is possible to create a non-uniform intensity distribution along the curve and yet preserving the phase profile, as observed in Fig. 1(d), and viceversa.

Variable optical current for any non-circular curve can easily be obtained by using, for example, the following constraint


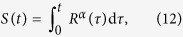


with *α* being a real number and *R*(*τ*) is given by the Superformula. Then, for uniform intensity distribution along the circuit, the optical current is proportional to 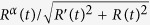. In Fig. 1(e–h) there are displayed the intensity and phase distributions of polymorphic beams (*α* = 2 and *l* = 34) in the form of: *Rose* **q** = (1.6, 1, 1.5, 2, 7.5, 12) Fig. 1(e), *modified-square* **q** = (1, 1, 15, 15, 15, 4) Fig. 1(f), *starfish* **q** = (10, 10, 2, 7, 7, 5) Fig. 1(g), and spiral **q** = (1, 1, 5, 5, 5, 10) Fig. 1(h). Here, *ρ*(*t*) = *ρ*_0_ is constant and *T* = 2*π* expect for the spiral in Fig. 1(h) where *ρ*(*t*) = *ρ*_0_*e*^0.2*t*^/40 and *T* = 6*π*.

**Figure 2:**
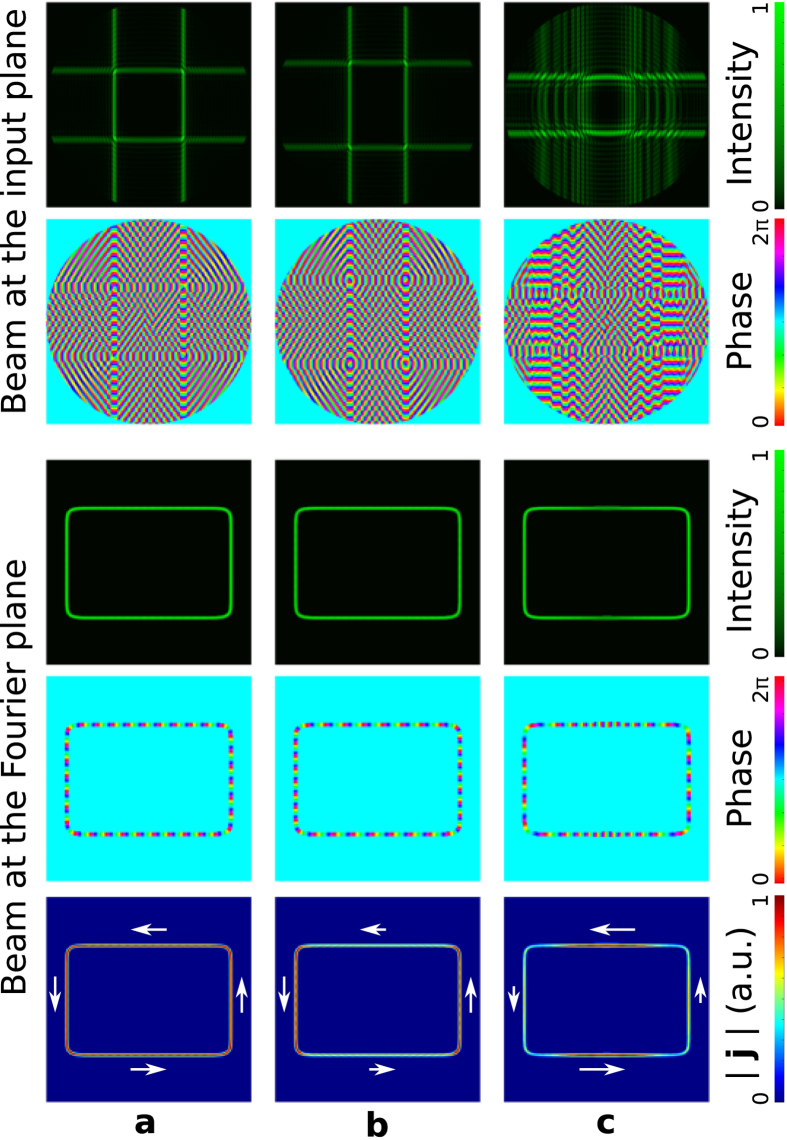
Setting different optical currents over the same rectangular circuit. Intensity and phase distributions of the rectangular polymorphic beam q = (1, 2/3, 15, 15, 15, 4) at the input (top panel) and at the Fourier plane (middle panel) for different phase of *g*(*t*). The focused beam has uniform intensity distribution in all the cases, but different phase distributions (*l* = 34): (**a**) Uniform phase obtained by using *S*(*t*) given by Eq. (9), and non-uniform phase by using Eq. (12) with *α* = 2 in (**b**) and *α* = −1 in (**c**). The distinct phase gradients yield different optical currents along the same circuit as displayed in the bottom row.

Note that well-defined circuits of different forms are preserved independently on the design of the optical current governed by the phase distribution along the curve. To illustrate this important fact, let us now consider a rectangle **q** = (1, 2/3, 15, 15, 15, 4) with uniform intensity but different phase distributions as displayed in Fig. 2. Specifically, in the case of Fig. 2(a) the phase is uniform (*S*(*t*) is given by Equation (9) and |∇Ψ| = constant), while in the case of Figs 2(b) and 1(c) the prescribed phase is variable (*S*(*t*) is given by Eq. (12)) by using *α* = 2 (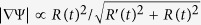) and *α* = −1 (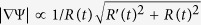), respectively. This example demonstrates that rather different optical currents can easily be created in the same circuit without altering its shape and size, see bottom row of Fig. 2.

### Holography for the experimental generation of polymorphic beams

**Figure 3:**
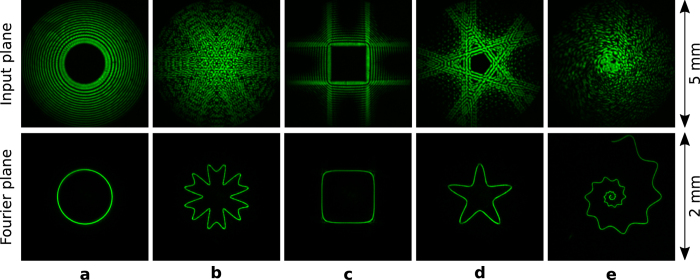
Experimental intensity distributions of the polymorphic beam (top panel) and of the corresponding optical circuits observed in the Fourier domain (bottom panel) for different curve shapes and topological charge *l* = 34: (**a**) circle, (**b**) rose, (**c**) modified-square, (**d**) starfish, and (**e**) spiral.

The complex field amplitude of the polymorphic beams studied in this work has been created in about 10 seconds by numerical calculation of the integral Eq. (2), programmed by us in Matlab. To experimentally generate the beam, it has been encoded (in ~5 s) as a phase-only hologram by using the technique reported in ref. 44. Here, we have used a programmable liquid-crystal spatial light modulator (SLM, Holoeye PLUTO, pixel size of 8 *μ*m) to display the hologram. As an example, the intensity distribution of several polymorphic beams measured by using a sCMOS camera (Hamamatsu, Orca Flash 4.0, 16-bit gray-level, pixel size of 6.5 *μ*m) are shown in the top row of Fig. 3. While, the corresponding intensity of the focused beams at the Fourier plane of a convergent lens (focal length of 15 cm) are presented in the bottom row of Fig. 3. These experimental results are in good agreement with the expected ones displayed in Fig. 1. Note that, in general, the curved beam observed in the Fourier plane can be directly encoded into the hologram instead of Eq. (2), however, the proposed method allows for focusing much more light along the circuit and better controlling of its phase and intensity distributions.

A static diffractive optical element^45^ can be used instead of a programmable SLM to display the hologram if needed. This is particularly important in the case of beam shaping for wavelengths outside of the visible spectrum and other types of coherent beams. For example, electron and x-ray vortex beams have been generated by using a hologram in the form of binary-amplitude mask^46^ or spiral plate^47^. To generate electron and x-ray polymorphic beams, as well as arbitrarily structured complex scalar fields, a proper arrangement of pinholes can be used as binary mask^48^.

### Optical current in action

The high intensity gradients of the optical circuits (Fourier transformed polymorphic beam) are important for single-shot material processing. Moreover, they create an attractive optical force on colloidal dielectric particles, with refractive index larger than the one of the surrounding medium, that can compensate the repulsive axial scattering force of light providing stable 3D optical trapping. On the other hand, the phase gradients along the curve redirect part of the light radiation pressure producing transverse optical forces. These forces are directly related to the optical current providing improved performance for laser micro-machining and ablation of materials as well as for driving colloidal dielectric micro-particles along the designed curves^26^,^49^.

**Figure 4:**
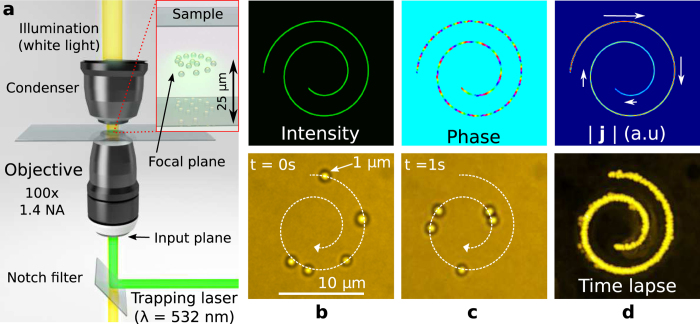
(**a**) Sketch of the optical trapping setup: The polymorphic beam is projected into the back aperture of a high numerical aperture (NA) microscope objective lens (see Methods) that focuses it over the sample (silica micro-spheres dispersed in water) in the form of Archimedean spiral. The particles are trapped within this curve and their motion is controlled by the optical current prescribed along the spiral circuit, see (**b**,**c**) and Supplementary Video 1. A time lapse image made by combining the recorded frames is shown in (**d**) and reveals the spiral trajectory of the particles. The optical current, displayed in the top row of (**d**), predicts the accelerating particle motion observed in the experiment, as expected. Note that the particles are optically manipulated far enough from the sample walls (25 *μ*m from the bottom glass coverslip), avoiding proximal hydrodynamical effects, as reported in ref. 26.

The action of the intensity gradient forces that confine colloidal dielectric particles within the circuit and the phase gradient force that propel them along it, is demonstrated here on the example of an Archimedean spiral circuit: *R*(*t*) = *ρ*_0_*t* and **q** = (1, 1, 0, 1, 1, 1) with *t* ∈ [*π*, 3*π*]. The corresponding polymorphic beam with phase distribution given by Eq. (12), with *α* = 2 and *l* = 34, is projected into the back aperture of a microscope objective lens as sketched in Fig. 4(a). The resulting focused beam traps in 3D several silica spheres of 1 *μ*m within the curve as observed in Fig. 4(b–d), see Methods and Supplementary video 1, far from the sample walls (25 *μ*m deep within the sample) thanks to the strong intensity gradient forces. The time-lapse image of the trapped particles displayed in Fig. 4(d) confirms the strong confinement revealing the shape of the light curve. The particles travel along the spiral towards its center according with the circuit shape and the created optical current displayed in the top row of Fig. 4(b–d). The motion is reversed in real time by setting the opposite *charge l* = −34 to avoid losing the particles when reaching the end points of the spiral, see Supplementary video 1. As it is expected the particles speed up when move out from the center of the spiral according with the optical current distribution.

## Discussion

We envision that the introduced concept of polymorphic beam inspired by Nature opens up promising perspectives. The polymorphic beam solves the problem of shaping laser light (without using iterative algorithms) in a large variety of forms creating closed or open circuits, but also allows for designing the optical current on demand. In contrast to the Gaussian vortex beams, these circuits are well-localized and their form as well as size are independent on the optical current flowing along as it has been demonstrated. This flexibility is an important achievement for all-optical transport and manipulation of small objects, laser micro-machining, study of micro-particle dynamics, etc. The versatility in the design of the polymorphic beam could be attractive for developing information encoding protocols for free-space laser communications based upon different shapes and/or topological charges^50^,^51^,^52^. The proposed approach can be used to design lattices of optical vortices required, for example, for fabrication of photonic crystals^53^. Indeed, as it is observed in Fig. 1 (in the input plane, first and second rows) for the case of the rose 1(e), starfish 1(g) and spiral 1(h) there exists a complex but well-structured vortex lattice field.

We underline that the curve shape can be specified by using other functions apart from the Superformula. Moreover, 2D circuits can be transformed into 3D ones by including the spherical phase term 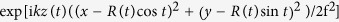 in the integral Eq. (2), where *z*(*t*) is the axial coordinate of the parametric 3D curve. As in the case of the Bessel beams^54^ the polymorphic scalar beam concept is extensible to the vector one. Polymorphic electron and x-ray beams can be created by specific holographic techniques based upon structured array of pinholes^48^, that paves the way to single-shot e-beam lithography adding the benefits of tailored phase gradients.

## Methods

The complex field amplitude given by expression Eq. (2) has been encoded for each case as a hologram and addressed into a programmable spatial light modulator (SLM, Holoeye PLUTO, pixel size of 8 *μ*m) as reported in ref. 49. The SLM was illuminated by a collimated laser beam (Laser Quantum, Ventus, *λ* = 532 nm, 1.5 W, linearly polarized) and the resulting beam relayed into the back aperture of the focusing lens: microscope objective lens (Olympus UPLSAPO, 1.4 NA, 100×). An oil immersion with *n* = 1.56 (Cargille Labs Series A) was used to mitigate the spherical aberration arising form the glass-water refractive index mismatch as considered in ref. 26. The power of the laser beam was ~170 mW at the back aperture of the objective lens. The particles were observed in bright-field mode, under white light illumination (LED, SugarCube Ultra), and recorded by a sCMOS camera (Hamamatsu, Orca Flash 4.0, 16-bit gray-level, pixel size of 6.5 *μ*m) at 30 frames per second. A Notch filter (Semrock, dichroic beamspliter for 532 nm) redirected the trapping beam into the objective lens, that prevents saturating the camera by backscattered laser light. The sample was enclosed into a chamber made by attaching two glass coverslip (thickness 0.17 mm). A double-sided Scotch tape (thickness about 100 *μ*m) was used as spacer between the coverslips. The 1 *μ*m silica spheres (Bang Labs) were filled into the sample cell directly from an aqueous solution (deionized water).

## Additional Information

**How to cite this article**: Rodrigo, J. A. and Alieva, T. Polymorphic beams and Nature inspired circuits for optical current. *Sci. Rep.* **6**, 35341; doi: 10.1038/srep35341 (2016).

## Supplementary Material

Supplementary Information

Supplementary Video S1
