# Supplementary material for: Polymorphic beams and Nature inspired circuits for optical current
Source: Sci Rep. 2016 Oct 13;6:35341. doi: 10.1038/srep35341 (PMC5062159; doi:10.1038/srep35341)
Supplement: Supplementary Information [file srep35341-s1.pdf]

Title: "Polymorphic beams and Nature inspired circuits for optical current"

Authors: Jose A. Rodrigo and Tatiana Alieva

Address: Universidad Complutense de Madrid, Facultad de Ciencias Fisicas,
Ciudad Universitaria s/n, Madrid 28040, Spain

Legend for the supplementary video:

Spiral circuit of optical current for confinement and switchable all-optical transport of dielectric micron-sized particles (silica).
